# Severe Paralytic Ileus and Pneumoperitoneum Without Perforation Following Epidural Morphine in a Young Post-gastrectomy Patient

**DOI:** 10.7759/cureus.87270

**Published:** 2025-07-04

**Authors:** Masaru Shimizu, Misao Yoshikawa, Mari Makio, Kakeru Okubo, Mao Kinoshita

**Affiliations:** 1 Department of Anesthesiology, Uji Tokushukai Medical Center, Uji, JPN; 2 Department of Anesthesiology, Kyoto Prefectural University of Medicine, Kyoto, JPN

**Keywords:** epidural morphine, gastrointestinal motility, paralytic ileus, pneumoperitoneum, postoperative ileus

## Abstract

Postoperative ileus (POI) is a relatively common complication of abdominal surgery that typically resolves with conservative treatment. However, in rare cases, it may progress to a more severe condition. We present the case of a 25-year-old man who developed severe POI and pneumoperitoneum without gastrointestinal perforation after receiving continuous epidural morphine following gastrectomy. Postoperative analgesia was maintained with the continuous epidural administration of morphine and ropivacaine. On postoperative day 2, the patient experienced worsening abdominal pain. Imaging studies revealed the presence of free intra-abdominal gas, a dilated intestinal tract, and portal venous gas. Emergency surgery was performed due to suspected intestinal necrosis; however, no perforation or obstruction was identified. The findings were attributed to increased intraluminal pressure and mucosal injury resulting from paralytic ileus. An ileal tube was inserted for decompression.

Following the second surgery, persistent abdominal distention prompted the discontinuation of epidural morphine. Subsequent improvement in bowl dilation was observed; the patient has remained in stable condition since. In this case, opioid-induced suppression of gastrointestinal motility likely contributed to the development of severe paralytic ileus and pneumoperitoneum. Although epidural morphine is generally considered safe, its potential impact on gastrointestinal function warrants caution. Regular monitoring of gastrointestinal motility and early intervention are essential during opioid use. This case report underscores the importance of risk assessment in postoperative pain management.

## Introduction

Effective postoperative pain management is essential for facilitating recovery, with opioids playing a central role in achieving adequate analgesia. Epidural morphine is widely used due to its reliable and prolonged pain-relieving effects [1,2]. However, opioids are known to inhibit gastrointestinal motility by activating μ-opioid receptors and suppressing acetylcholine release, which may lead to complications such as paralytic ileus [3]. Mild postoperative ileus (POI) is relatively common but rarely leads to non-perforated pneumoperitoneum [4,5]. Idiopathic pneumoperitoneum, a rare condition without an identifiable cause, is typically managed conservatively [5]. The occurrence of severe paralytic ileus accompanied by non-perforated pneumoperitoneum in a healthy young individual is exceedingly uncommon and carries significant implications for clinical decision-making.

## Case presentation

**Figure 1:**
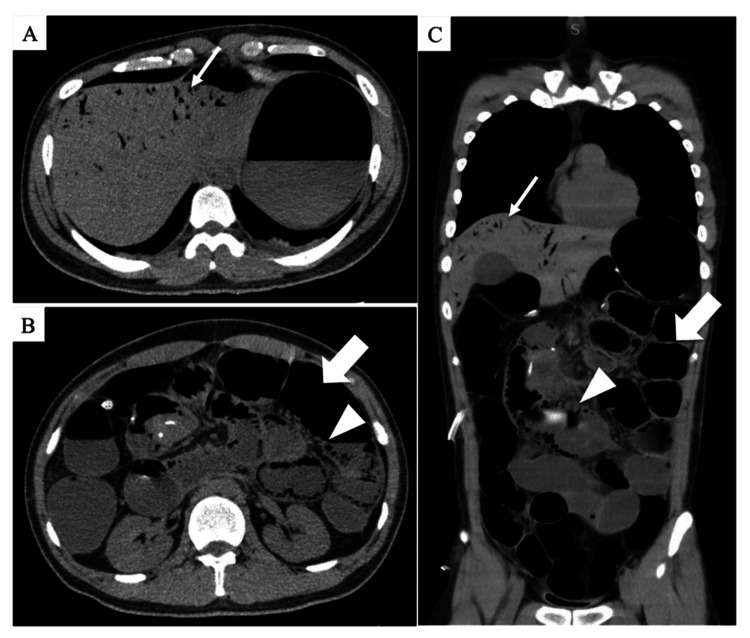
Abdominal CT scan obtained prior to the second surgery. Axial CT scan images showing bowel distension (arrow) (A, C) and emphysema of the intestinal wall (arrowhead) and portal vein (narrow arrow) (B, C). These findings suggest a significant increase in intestinal pressure and mucosal injury, strongly indicating the possibility of intestinal necrosis and severe ischemia. CT: computed tomography

The patient was a 25-year-old man, measuring 174 cm in height and weighing 72 kg, who presented to the hospital with complaints of abdominal pain and melena. The endoscopic evaluation revealed a bleeding gastric ulcer. Subsequent histopathological examination confirmed the presence of gastric cancer. Gastrectomy was scheduled accordingly. The patient's past medical history was unremarkable, and no psychiatric disorders were reported in the family history. A proton pump inhibitor was administered orally prior to the surgery. On admission, laboratory tests showed anemia, with a hemoglobin level of 10.2 mg/dL. Computed tomography (CT) demonstrated no evidence of ascites, masses, or metastatic nodules. The patient underwent robot-assisted distal gastrectomy under combined general and epidural anesthesia. The operative time was six hours and 46 minutes, with a total anesthesia duration of eight hours and 16 minutes. Intraoperative analgesia included intravenous fentanyl (300 μg), a continuous remifentanil infusion (0.1-0.16 μg/kg/min), and 2 mg of morphine administered via epidural injection. Postoperative analgesia was maintained with a continuous epidural infusion of morphine at 4 mg/day combined with 0.2% ropivacaine. On postoperative day 2, the patient developed worsening abdominal pain. Consequently, blood tests were performed, and a CT scan was obtained. Laboratory tests revealed a white blood cell count of 8,900/mm³ and a C-reactive protein level of 7.67 mg/dL. Renal, hepatic, and electrolyte profiles were within normal limits. CT imaging demonstrated the presence of free intra-abdominal gas, marked intestinal dilation, and portal venous gas (Figure 1).

**Figure 2:**
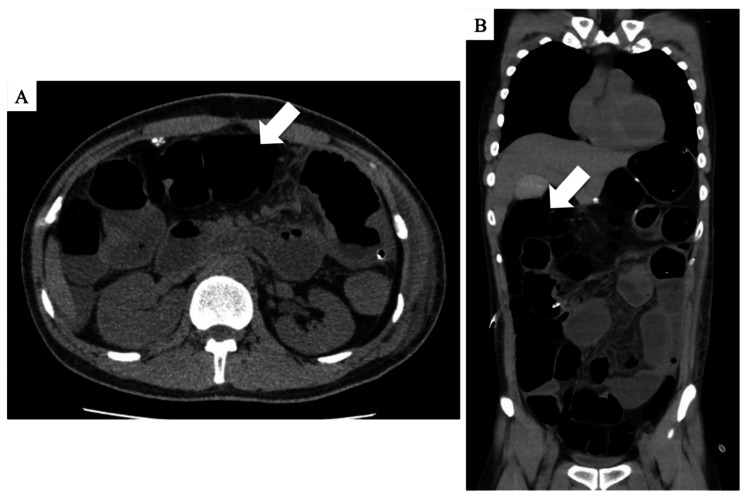
Abdominal CT scan obtained two days after the second surgery. Axial CT imaging showing persistent bowel distension (arrow), although no free intraperitoneal air is observed. These findings were interpreted as ongoing intestinal dilatation due to postoperative intestinal paralytic ileus. CT: computed tomography

The abdominal pain intensified to an unbearable degree. Emergency laparotomy was undertaken, as early diagnosis and treatment of intestinal necrosis are critical for improving prognosis. The differential diagnoses included anastomotic leakage, intestinal perforation, intestinal necrosis, mesenteric vein thrombosis, and gastrointestinal emphysema. Based on clinical and radiologic findings, intestinal necrosis was strongly suspected. Intraoperative findings revealed marked dilation of the small intestine, with emphysematous changes observed in the intestinal wall and mesentery of the lower small intestine. No gross perforation was identified. These findings were interpreted as mucosal injury caused by elevated intraluminal pressure secondary to paralytic ileus. Approximately 2.3 L of intestinal contents were aspirated to achieve decompression. An ileal tube was inserted nasally, with the tip advanced into the afferent loop. Postoperatively, epidural infusion of morphine (4 mg/day) and 0.2% ropivacaine was continued. However, abdominal distention persisted. Two days after the second surgery, repeat CT imaging demonstrated persistent and extensive small bowel dilation (Figure 2).

**Figure 3:**
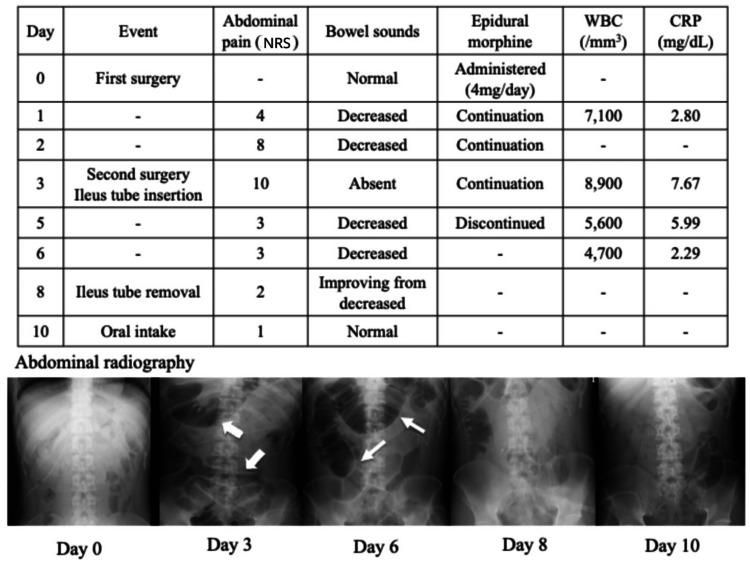
Timeline of the postoperative clinical course timeline and representative abdominal radiographic findings. The timeline illustrates key postoperative periods (days 0-10), including the timing of epidural morphine administration and discontinuation, the presence of abdominal pain, bowel sound activity, and inflammatory response parameters. On postoperative day 2, physical examination revealed decreased bowel sounds, supporting the diagnosis of paralytic ileus. Epidural morphine administration was discontinued, after which abdominal symptoms showed gradual improvement. Follow-up abdominal radiography demonstrated a reduction in bowel dilatation. Plain abdominal radiography obtained on postoperative day 3 (following the second surgery) revealed extensive intestinal dilatation with intraluminal gas (arrow). On postoperative day 6, persistent and prominent intestinal dilatation was observed (narrow arrow). NRS: Numerical Rating Scale; WBC: white blood cell; CRP: C-reactive protein

Pantothenic acid and a prostaglandin F2α preparation, both known to promote gastrointestinal motility, were administered. Epidural morphine was subsequently discontinued, and analgesia was continued with 0.2% ropivacaine alone. Improvement in abdominal symptoms was observed three days after the second surgery, and abdominal radiographs confirmed reduced bowel dilatation. The epidural catheter was removed five days after the second surgery. The patient gradually resumed oral intake and was discharged 14 days after the second surgery. A timeline of clinical events is presented in Figure 3.

## Discussion

We report a rare case of a young man who developed paralytic ileus following the postoperative administration of epidural morphine. The condition progressed despite conservative treatment and ultimately resulted in pneumoperitoneum without gastrointestinal perforation. Paralytic ileus is a common postoperative complication associated with abdominal surgery. Postoperative sympathetic activation and opioid administration are known to inhibit intestinal motility, leading to the stagnation of intestinal contents for 72-96 hours after surgery [6]. In addition, the inflammatory response to intraoperative intestinal manipulation may further impair gastrointestinal function. Infiltration of immune cells into the intestinal wall and the subsequent release of inflammatory cytokines contribute to postoperative smooth muscle dysfunction and impaired motility [7]. The incidence of POI following abdominal surgery is estimated at approximately 14% [4]. Although a higher prevalence has been reported among male patients, the influence of sex-related factors on POI remains unclear. The mortality rate associated with ileus ranges from 2% to 10% [8]. In the United States, the annual healthcare costs attributed to ileus have been estimated to exceed $750 million, highlighting the clinical and economic burden of this condition [9]. The etiology of ileus is multifactorial, involving a complex interplay of gastrointestinal tract distension, fluid and electrolyte imbalances, opioid use, neurohormonal dysregulation, and inflammatory responses [10]. Known risk factors for POI include open surgical procedures, prolonged operative duration, and extended hospital stays [11]. Although Enhanced Recovery After Surgery protocols have been widely implemented to mitigate postoperative complications, the incidence of POI remains substantial [12].

Epidural analgesia is considered a standard and effective approach for postoperative pain management. Compared with intravenous opioid administration, it has been associated with earlier recovery of gastrointestinal function and reduced systemic opioid requirements [1,2]. In the majority of patients, POI improves with supportive measures, including fasting, intravenous fluid administration, prokinetic agents, and nasogastric decompression [13]. However, the effectiveness of motility-enhancing medications may be limited in patients with severe disease, extensive intestinal paralysis, or compromised general condition [14]. In such cases, these agents must be administered with caution due to the potential risk of intestinal perforation. When drug-induced ileus is suspected, discontinuation of the causative drug is typically effective in reversing the condition. If POI fails to resolve with conservative treatment, endoscopic or surgical intervention should be considered [15]. Idiopathic pneumoperitoneum, an extremely rare condition without an identifiable cause, is typically managed conservatively in the absence of signs indicating gastrointestinal perforation [5].

Epidural morphine was the first opioid approved by the Food and Drug Administration for spinal administration and remains widely utilized in clinical practice [16]. As a hydrophilic agent, morphine exhibits a slower onset and prolonged duration of action compared with lipophilic opioids. It diffuses cephalad within the cerebrospinal fluid, exerting its analgesic effects through action on the medulla oblongata and brainstem [17]. Epidural morphine is approximately 5-10 times more potent than intravenous morphine and is typically administered in bolus doses of 30-100 g/kg or via continuous infusion at rates of 0.2-0.4 mg/hour. For opioid-naive patients, an initial epidural dose of 3.5-7.5 mg/day is generally recommended [18]. Compared with intravenous or intramuscular administration, epidural morphine exerts a lesser inhibitory effect on colonic electromyographic activity [19]. As a result, it is often considered beneficial for facilitating the earlier postoperative recovery of gastrointestinal function [20]. However, opioid-induced constipation remains a significant contributor to the development of POI. Morphine impairs gastrointestinal motility by activating μ-opioid receptors within the myenteric plexus, thereby inhibiting acetylcholine release and suppressing intestinal peristalsis [3].

In this case, epidural morphine was administered within the recommended dosage range; however, the cumulative suppression of gastrointestinal motility was considered to have contributed to the development of POI. This highlights the need for regular monitoring of bowel function in patients receiving postoperative opioids. If signs of impaired gastrointestinal motility emerge, prompt discontinuation or modification of opioid therapy is essential. Although the epidural route is generally regarded as safer than systemic administration, this case demonstrates that significant adverse effects on gastrointestinal function may still occur. Accordingly, perioperative pain management should be guided by continuous assessment of patient-specific risk factors and close clinical observation.

## Conclusions

Epidural morphine remains an effective modality for perioperative analgesics. However, its inhibitory effects on gastrointestinal motility may result in serious complications such as paralytic ileus. In the present case, epidural morphine was the most strongly suspected factor contributing to intestinal dysfunction, although a definitive causal relationship has not been established. However, early recognition and timely intervention are critical when opioid-related bowel dysfunction is suspected. In the future, the adoption of multimodal analgesic strategies, consideration of opioid-sparing alternatives, and development of individualized pain management plans based on patient-specific risk factors will be essential in optimizing outcomes while minimizing adverse effects.
